# Supplementary material for: Attention modulates neural representation to render reconstructions according to subjective appearance
Source: Commun Biol. 2022 Jan 11;5:34. doi: 10.1038/s42003-021-02975-5 (PMC8752808; doi:10.1038/s42003-021-02975-5)
Supplement: Supplementary file 2 — Supplementary Information [file 42003_2021_2975_MOESM2_ESM.pdf]

Supplementary Information

Attention modulates neural representation to render reconstructions according to subjective appearance

Tomoyasu Horikawa[1*], Yukiyasu Kamitani[1,2*]

1. Department of Neuroinformatics, ATR Computational Neuroscience Laboratories, Kyoto, Japan
2. Graduate School of Informatics, Kyoto University, Kyoto, Japan
* Correspondence: horikawa.t@gmail.com, kamitani@i.kyoto-u.ac.jp

Supplementary Figures

Supplementary Figure 1 Feature decoding and reconstruction results for single-image trials.
Supplementary Figure 2 Examples of reconstructed images for attention trials.
Supplementary Figure 3 Peak shifts at visual subareas for individual subjects.
Supplementary Figure 4 Identification accuracy for individual subjects.
Supplementary Figure 5 Cohen's *d* of identification accuracy for individual subjects.
Supplementary Figure 6 Amplitude of decoded features in attention trials for individual subjects.
Supplementary Figure 7 Cohen's *d* of amplitude differences between the attended and unattended feature sets at visual subareas for individual subjects.

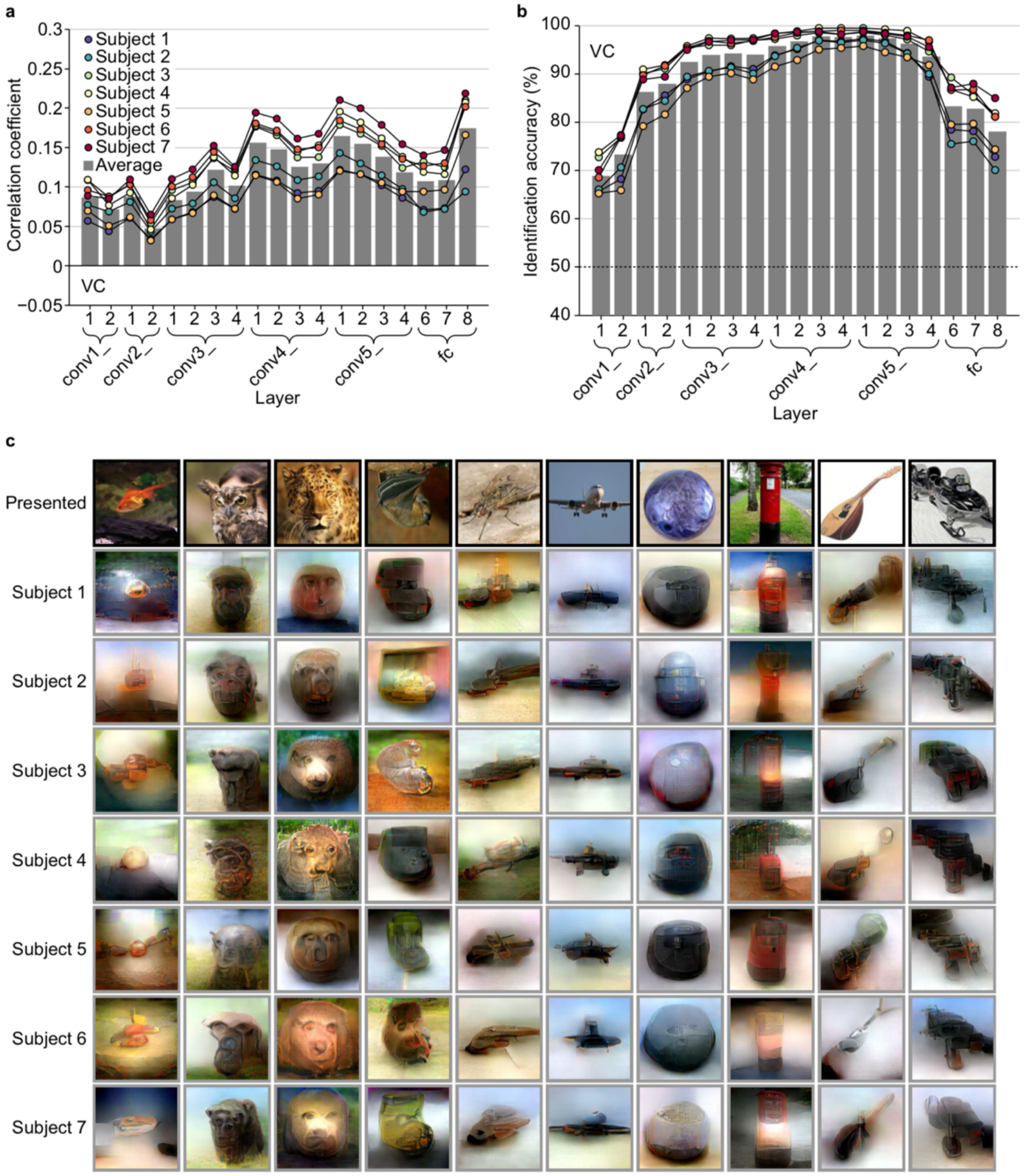

Supplementary Figure 1 Feature decoding and reconstruction results for single-image trials. a Feature decoding accuracy for single-image trials. A decoding accuracy was evaluated by calculating a Pearson correlation coefficient between a pattern of decoded feature values and a pattern of image feature values computed from presented images for each sample (decoded from the visual cortex [VC]; normalized by the values in training images for each unit). Correlation coefficients were averaged across samples from the single-image trials (a total of 160 trials for each subject, colored dots), and the mean correlations averaged across subjects (gray bars)

are shown for each layer of the VGG19 model. For all subjects and DNN layers, the feature decoding accuracies were significantly higher than the chance (one-sided *t*-test, $p < 0.01$, Bonferroni correction by the numbers of DNN layers and subjects). **b** Pair-wise image identification accuracy for single-image trials. Identification accuracy obtained by the pair-wise identification analysis is shown for each layer of the VGG19 model (decoded from VC; normalized by the values in training images for each unit; chance level, 50%; see Methods: "Identification analysis"). In the analysis, correlation coefficients were calculated between a pattern of decoded features and patterns of image features of two candidate images (one for true [presented], and the other for false), and the image with a higher correlation coefficient was selected as the predicted image. For each sample, pair-wise identification was performed for all pairs between one true image and the other nine false images used in the test session (nine pairs for each sample). The accuracy of each sample was defined by the proportions of correct identification. For all subjects and DNN layers, the identification accuracies were significantly higher than the chance (one-sided *t*-test, $p < 0.01$, Bonferroni correction by the numbers of DNN layers and subjects). Conventions are the same with Supplementary Figure 1a. **c** Examples of reconstructed images from single-image trials. The reconstructed images produced from samples of each of the single-image trials are shown for seven subjects (decoded from VC). Conventions are the same with Fig. 2b. It is noteworthy that even though the average correlation coefficient of feature decoding accuracy was not high (in the range of 0.05 to 0.2, Supplementary Figure 1a), the identification accuracy of the viewed images reached more than 80% for most DNN layers, and the quality of the reconstructed images reached a level sufficient to identify objects (84.7%, averaged across subjects; Fig. 2d). These high performances of the identification and reconstruction analyses indicate that correlations around such a range are not negligible. Furthermore, when we took a closer look at the relationship between the reconstruction accuracy from single-image trials (Fig. 2d) and the feature decoding accuracy (Supplementary Figure 1a and b), we found that a group of subjects with relatively high decoding accuracy (e.g., Subject 3, 4, 6, and 7) performed better in the reconstruction analysis of seen images, and vice versa (see Subject 1, 2, and 5 for the case with relatively low accuracy). This positive relationship between performances of feature decoding and image reconstruction may confirm that decoded features with high accuracy can be interpreted as a reliable measure of the visual information represented in the brain, and it can be inferred that the decoding accuracy/features obtained from the attention condition reflect visual information modulated by attention.

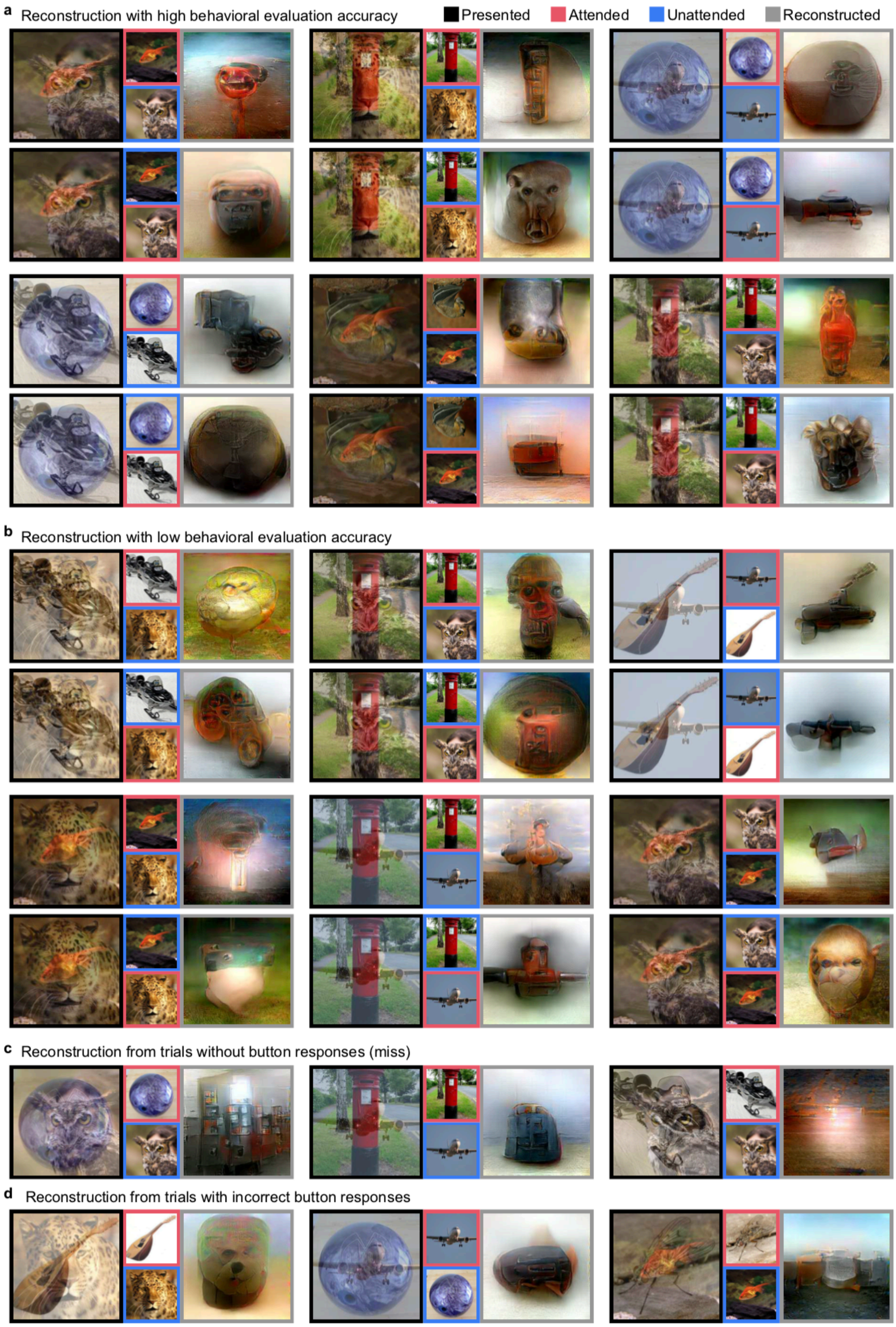
a Reconstruction with high behavioral evaluation accuracy
Presented
Attended
Unattended
Reconstructed
b Reconstruction with low behavioral evaluation accuracy
c Reconstruction from trials without button responses (miss)
d Reconstruction from trials with incorrect button responses

Supplementary Figure 2 Examples of reconstructed images for attention trials. a Examples of attended image reconstructions with high behavioral rating accuracies. Reconstructed images with relatively high rating accuracies (higher than 80%) are shown. Conventions are the same with Fig. 2a. **b** Examples of attended image reconstructions with low rating accuracies. Reconstructed images with relatively low rating accuracies (lower than 60%) are shown. Failures of attended image reconstructions were categorized into clutter images, mixtures of two superimposed images, or images more similar to unattended images. **c** Reconstructed images from samples for trials without button responses. Reconstructed images obtained from samples for miss trials, in which subjects missed a button-press to indicate correct recognition of target images, are shown. The reconstructions from these miss trials tended to be not similar to either of the two superimposed images. **d** Reconstructed images from samples for trials with incorrect button responses. Reconstructed images obtained from samples for error trials, in which subjects incorrectly pressed a button to indicate target images, are shown. The reconstructions from these error trials sometimes produced images judged to be similar to non-target (or instructed to be unattended) images.

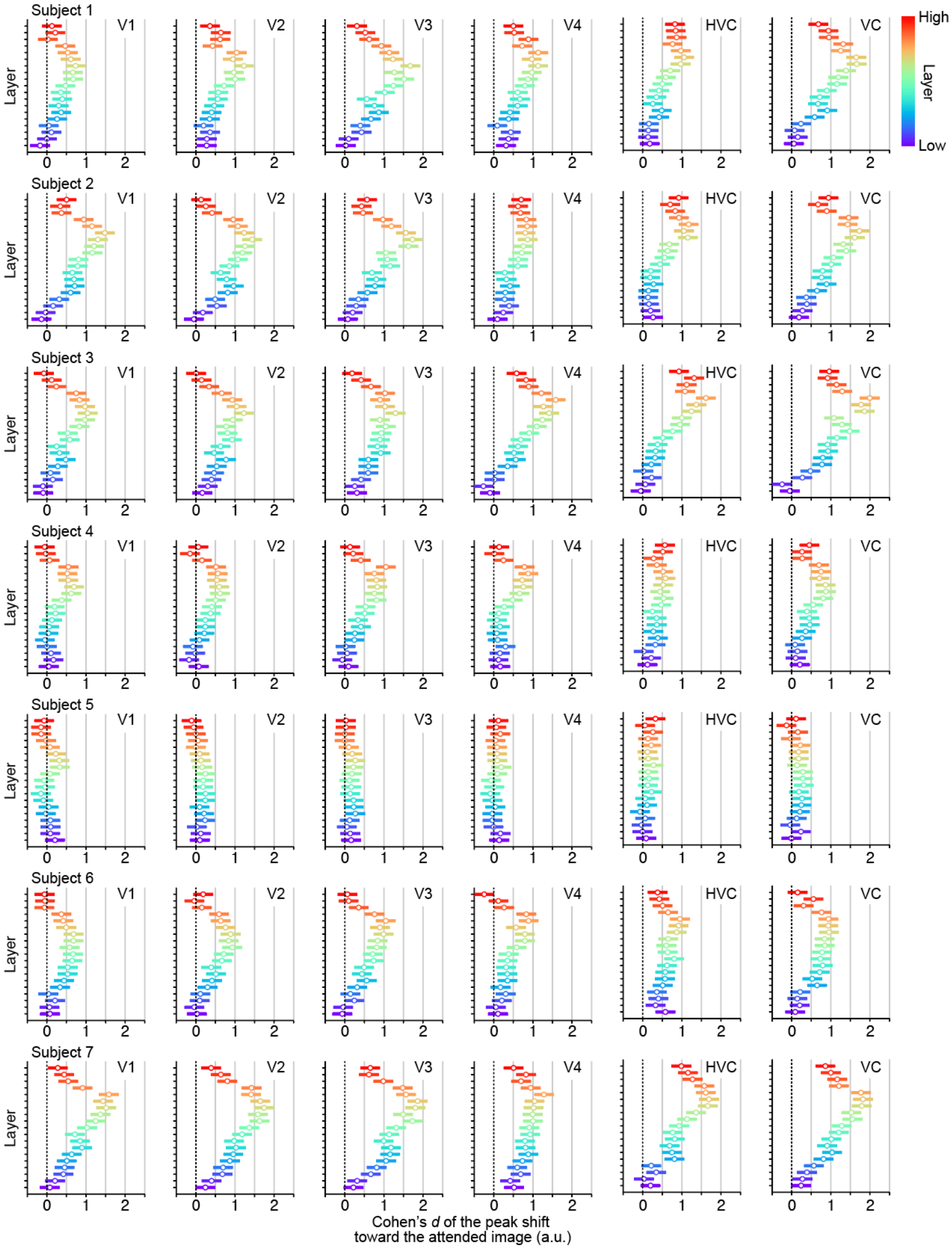

Supplementary Figure 3 Peak shifts at visual subareas for individual subjects. Conventions are the same with Fig. 3d. Subjects whose reconstructions from attention trials were evaluated highly (e.g., Subject 1–3 and 7; cf., Fig. 2c) tended to specifically show greater biases in decoded feature patterns. The results showed that the significant effect of attentional modulations observed in four of the initial five subjects

(Subject 1–4) were replicated with the two additionally collected subjects (Subject 6 and 7).

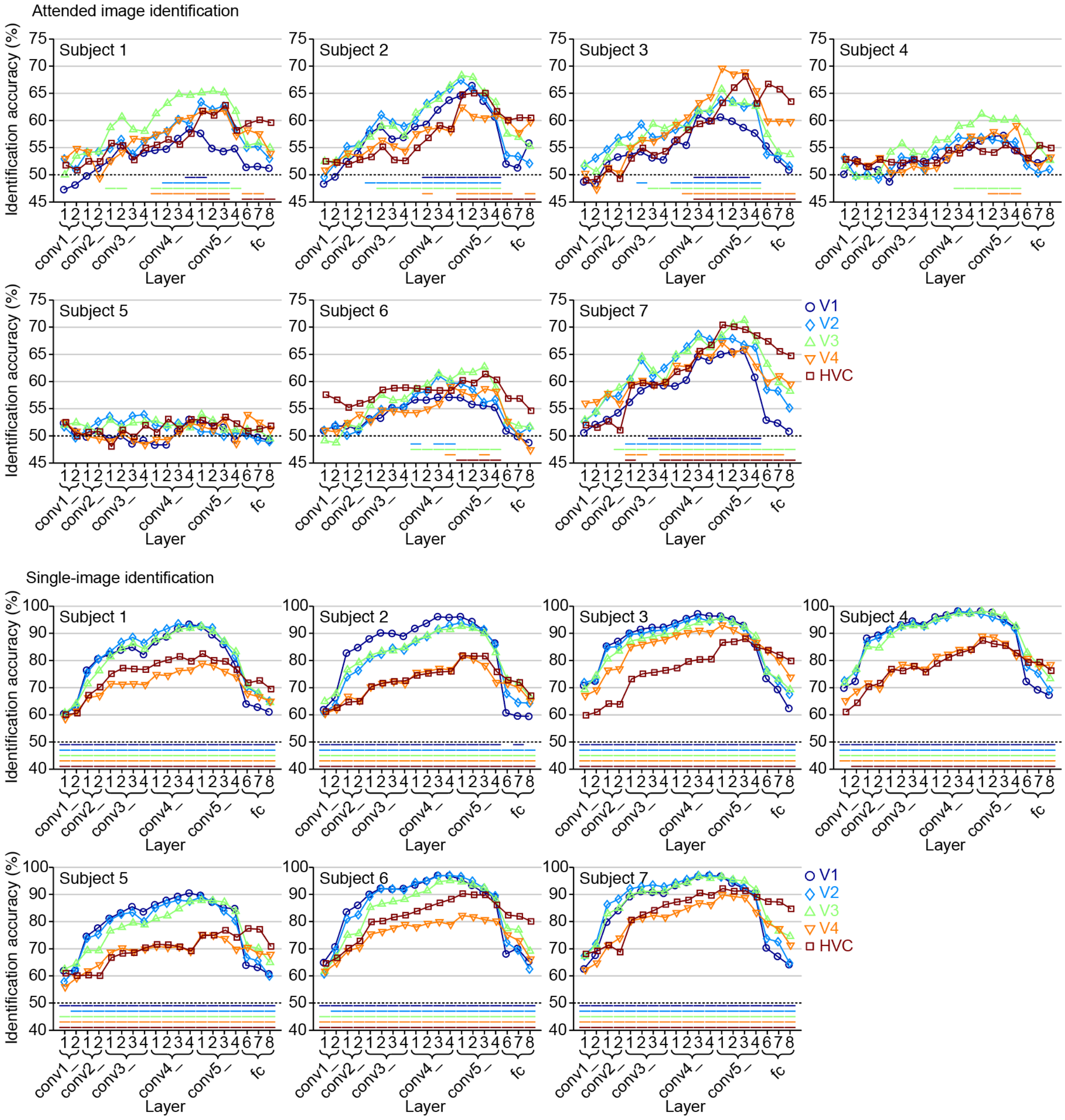

Supplementary Figure 4 Identification accuracy for individual subjects. Mean identification accuracies of individual subjects are shown for all combinations of individual visual subareas and DNN layers. Conventions are the same with Fig. 4b. The results showed relatively greater variability among subjects in the accuracies of the attended image identification than those of the single-image identification, possibly due to the individual differences in the ability to direct their selective attention. Differences in brain areas that showed high attended image identification accuracies might be attributable to differences in their strategies for attention, as we did not explicitly provide specific strategies for their attempt of attention.

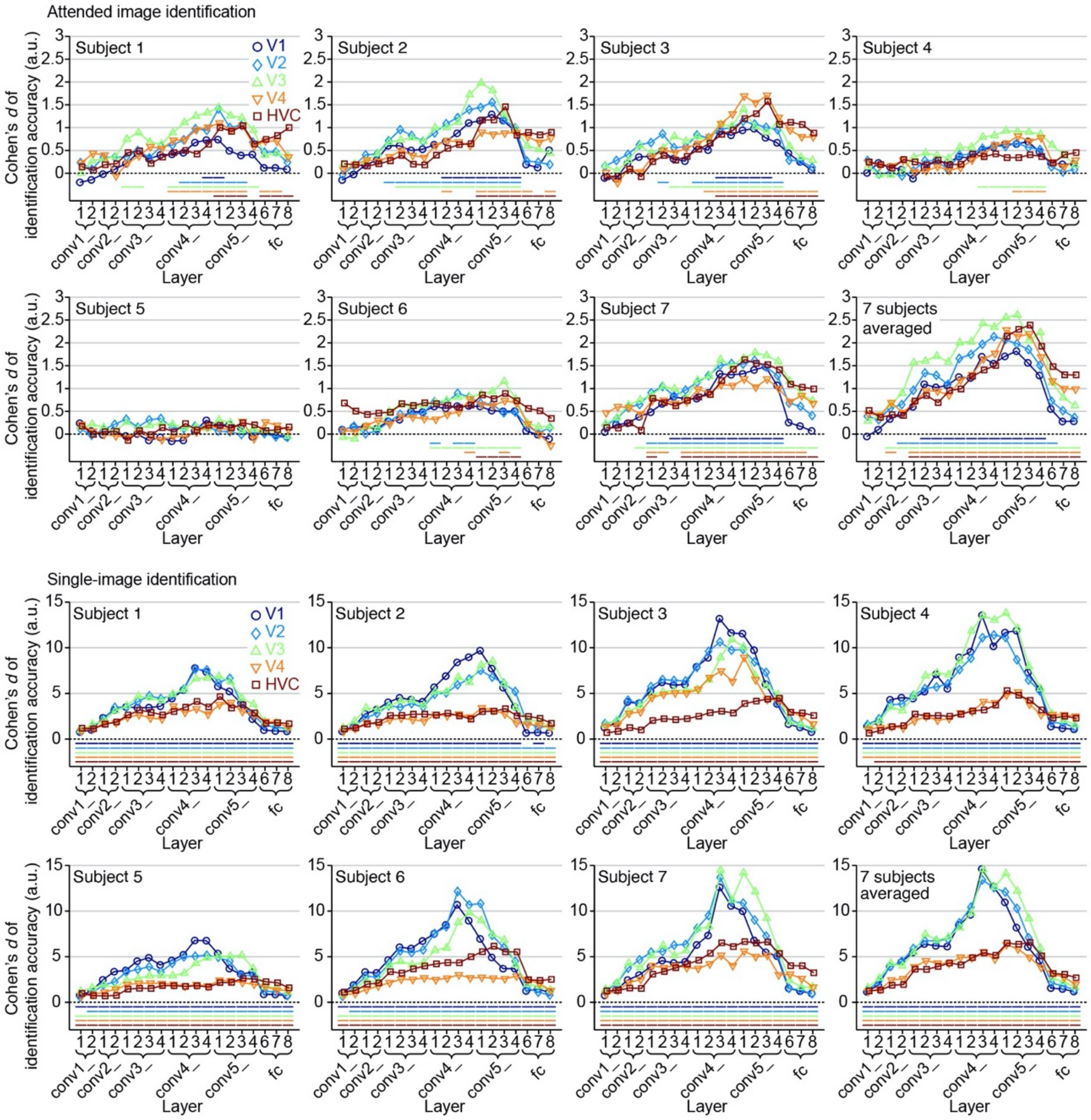

Supplementary Figure 5 Cohen's *d* of identification accuracy for individual subjects. For each combination of brain areas and DNN layers, the Cohen's *d* of the identification accuracy was calculated by first subtracting 50 (chance level) from the identification accuracy averaged for each pair (45 pairs) and then by normalizing the accuracy by the standard deviation across pairs. Colored lines underneath the data indicate the results of statistical tests that are the same with those shown in Fig. 4b and Supplementary Figure 4. Conventions are the same with Supplementary Figure 4. On average, the results showed medium-to-large effect sizes in the attention condition, indicating the robustness of the attentional modulations on decoded features.

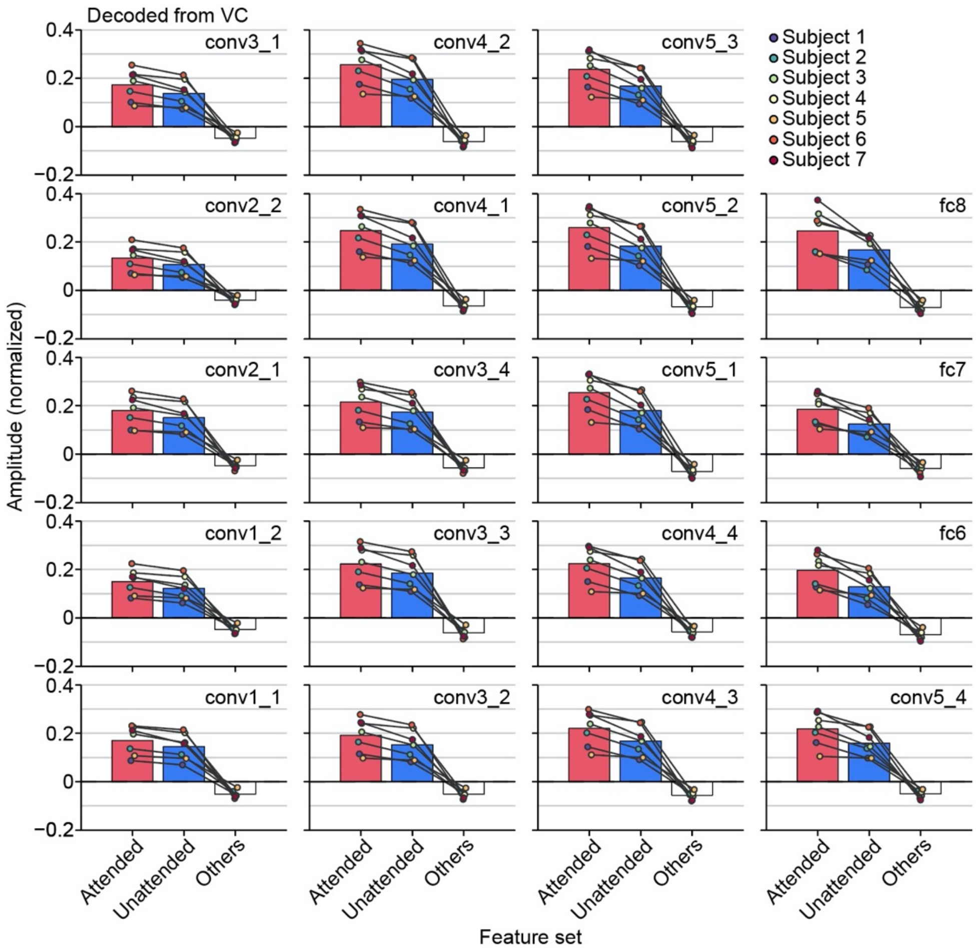

Supplementary Figure 6 Amplitude of decoded features in attention trials for individual subjects. Mean amplitudes averaged across units, trials, and image pairs for each subject are shown for the three types of feature sets (decoded from VC). Dots and bars indicate mean amplitudes for individual subjects and their average, respectively.

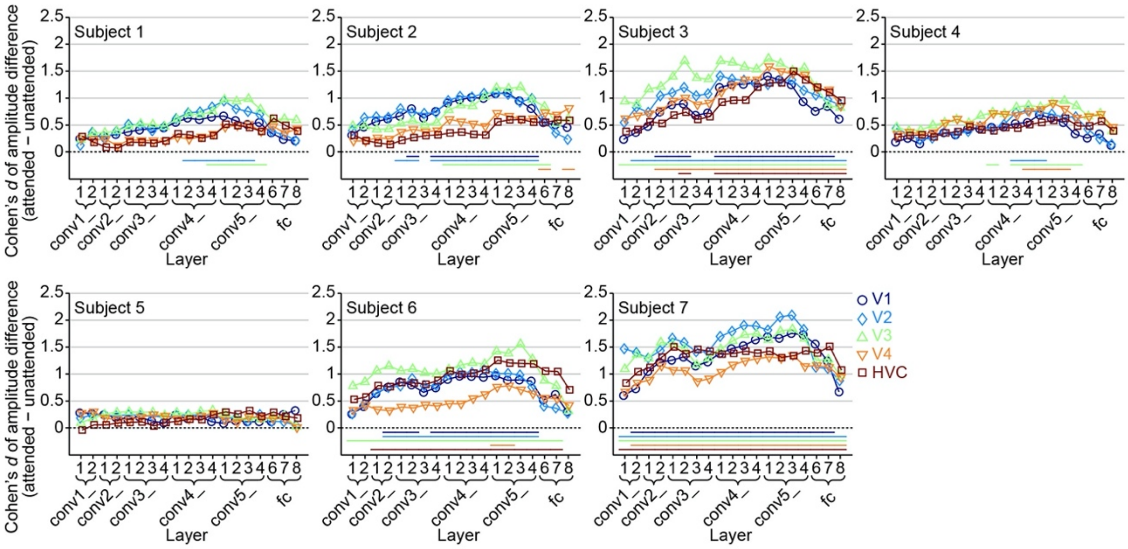

Supplementary Figure 7 Cohen's *d* of amplitude differences between the attended and unattended feature sets at visual subareas for individual subjects. The effect sizes (Cohen's *d*) of amplitude differences between the feature sets of attended and unattended images of individual subjects are shown for all combinations of areas and layers. Conventions are the same with Fig. 5c.
