# Supplementary material for: Attention modulates neural representation to render reconstructions according to subjective appearance
Source: Commun Biol. 2022 Jan 11;5:34. doi: 10.1038/s42003-021-02975-5 (PMC8752808; doi:10.1038/s42003-021-02975-5)
Supplement: Supplementary file 3 — Description of Additional Supplementary Files [file 42003_2021_2975_MOESM3_ESM.pdf]

Description of Additional Supplementary Files

File name: Supplementary Movie 1

Description: *Reconstructions of attended images*. The iterative optimization process is shown for reconstructions from attention trials (the last 80 steps of a total of 200 optimization steps; See Methods: "Visual image reconstruction analysis"; left, presented image; center, attended or unattended image; red frame, attended image; right, reconstructed image; cf., Fig. 2; https://youtu.be/iJAF8d7d9dc).
