# Supplementary material for: Attention modulates neural representation to render reconstructions according to subjective appearance
Source: Commun Biol. 2022 Jan 11;5:34. doi: 10.1038/s42003-021-02975-5 (PMC8752808; doi:10.1038/s42003-021-02975-5)
Supplement: Supplementary file 5 — Reporting Summary [file 42003_2021_2975_MOESM5_ESM.pdf]

nature research

Corresponding author(s): Tomoyasu Horikawa, Yukiyasu Kamitani

Last updated by author(s): Nov 21, 2021

Reporting Summary

Nature Research wishes to improve the reproducibility of the work that we publish. This form provides structure for consistency and transparency in reporting. For further information on Nature Research policies, see our Editorial Policies and the Editorial Policy Checklist.

Statistics

For all statistical analyses, confirm that the following items are present in the figure legend, table legend, main text, or Methods section.

n/a	Confirmed	
☐	☒	The exact sample size (*n*) for each experimental group/condition, given as a discrete number and unit of measurement
☐	☒	A statement on whether measurements were taken from distinct samples or whether the same sample was measured repeatedly
☐	☒	The statistical test(s) used AND whether they are one- or two-sided<br>*Only common tests should be described solely by name; describe more complex techniques in the Methods section.*
☐	☒	A description of all covariates tested
☐	☒	A description of any assumptions or corrections, such as tests of normality and adjustment for multiple comparisons
☐	☒	A full description of the statistical parameters including central tendency (e.g. means) or other basic estimates (e.g. regression coefficient) AND variation (e.g. standard deviation) or associated estimates of uncertainty (e.g. confidence intervals)
☐	☒	For null hypothesis testing, the test statistic (e.g. *F*, *t*, *r*) with confidence intervals, effect sizes, degrees of freedom and *P* value noted<br>*Give P values as exact values whenever suitable.*
☒	☐	For Bayesian analysis, information on the choice of priors and Markov chain Monte Carlo settings
☒	☐	For hierarchical and complex designs, identification of the appropriate level for tests and full reporting of outcomes
☐	☒	Estimates of effect sizes (e.g. Cohen's *d*, Pearson's *r*), indicating how they were calculated

Our web collection on statistics for biologists contains articles on many of the points above.

Software and code

Policy information about availability of computer code

Data collection	fMRI data were collected in experiments controlled by custom code written with Psychtoolbox on MATLAB R2017b.
Data analysis	Analyses were performed with MATLAB, python2.7, FMRIPREP (version 1.2.1), FSL (version 5.0.9), AFNI (version 16.2.07), FreeSurfer (version 6.0.1), and ANTs (version 2.1.0).

For manuscripts utilizing custom algorithms or software that are central to the research but not yet described in published literature, software must be made available to editors and reviewers. We strongly encourage code deposition in a community repository (e.g. GitHub). See the Nature Research guidelines for submitting code & software for further information.

Data

Policy information about availability of data

All manuscripts must include a data availability statement. This statement should provide the following information, where applicable:

- Accession codes, unique identifiers, or web links for publicly available datasets
- A list of figures that have associated raw data
- A description of any restrictions on data availability

The raw and preprocessed fMRI data for seven subjects are available from open data repository (raw data in OpenNeuro: https://openneuro.org/datasets/ds001506/versions/1.3.1; https://openneuro.org/datasets/ds003430; preprocessed data in figshare: https://doi.org/10.6084/m9.figshare.13474629).

Field-specific reporting

Please select the one below that is the best fit for your research. If you are not sure, read the appropriate sections before making your selection.

- [x] Life sciences
- [ ] Behavioural & social sciences
- [ ] Ecological, evolutionary & environmental sciences

For a reference copy of the document with all sections, see nature.com/documents/nr-reporting-summary-flat.pdf

Life sciences study design

All studies must disclose on these points even when the disclosure is negative.

Sample size	The original sample size was chosen on the basis of previous fMRI studies with similar experimental designs (n = 5), and then we further collected data from additional two subjects (Subjects 6 and 7) following the request by the editor through the revision. The first three subjects (Subjects 1–3) were the same as those in a previous study (Shen et al., 2019). For these subjects, we reused a subset of previously published data (data for the training session, which was originally referred to as "training natural image session" of the "image presentation experiment"; available from https://openneuro.org/datasets/ds001506/versions/1.3.1), while newly collecting additional data (data for the test session). For the last four subjects (Subjects 4–7), we newly collected a whole dataset (data for the training session and the test session).
Data exclusions	No data were excluded.
Replication	Results from multiple subjects and trials can be considered as replications of the analysis. Using fMRI data of initially collected five tested subjects, the main findings were independently replicated from four subjects with multiple successful trials for each subject. Furthermore, at the request of the editor and reviewers during the revision, we have additionally collected data from two more subjects and confirmed the replicability of the main findings with those new subjects.
Randomization	The full fMRI dataset was collected from individual subjects (within-subject design), and no subject randomization was performed.
Blinding	Blinding was not relevant, because no randomization was done.

Reporting for specific materials, systems and methods

We require information from authors about some types of materials, experimental systems and methods used in many studies. Here, indicate whether each material, system or method listed is relevant to your study. If you are not sure if a list item applies to your research, read the appropriate section before selecting a response.

Materials & experimental systems

n/a	Involved in the study	
x		Antibodies
x		Eukaryotic cell lines
x		Palaeontology and archaeology
x		Animals and other organisms
	x	Human research participants
x		Clinical data
x		Dual use research of concern

Methods

n/a	Involved in the study	
x		ChIP-seq
x		Flow cytometry
	x	MRI-based neuroimaging

Human research participants

Policy information about studies involving human research participants

Population characteristics	Seven healthy subjects with normal or corrected-to-normal vision participated in our experiments: Subject 1 (male, age 34–36), Subject 2 (male, age 23–24), Subject 3 (female, age 23–24), Subject 4 (male, age 22–23), Subject 5 (male, age 27–29), Subject 6 (female, age 27–28), and Subject 7 (male, age 30–31).
Recruitment	Subjects were recruited for their ability to participate into multiple fMRI session, in which each session can take at most 2 hours. All subjects had considerable experience participating in fMRI experiments, and were highly trained. Thus, these characteristics of the subjects may contribute to guarantee the quality of the data.
Ethics oversight	The study protocol was approved by the Ethics Committee of ATR.

Note that full information on the approval of the study protocol must also be provided in the manuscript.

Magnetic resonance imaging

Experimental design

Design type	Block design
Design specifications	All seven subjects participated into two types of experimental sessions: a training session and a test session. Data from each subject were collected over multiple scanning sessions. On each experimental day, one consecutive session was conducted for a maximum of 2 hours. Subjects were given adequate time for rest between runs (every 7–10 min) and were allowed to take a break or stop the experiment at any time. The training and test sessions both consisted of 24 and 16 separate runs, respectively. Each run comprised 55 trials (7 min 58 s for each run). Each trial was 8-s long with no rest period between trials. Additional 32- and 6-s rest periods were added to the beginning and end of each run, respectively. The whole training session was repeated five times.
Behavioral performance measures	The behavioral performance during attention trials in the test session: percentages of correct, error, and miss trials among a total of 720 attention trials; 99.4%, 0.6%, and 0% for Subject 1; 98.8%, 0.6%, and 0.7% for Subject 2; 97.4%, 0.8%, and 1.8% for Subject 3; 99.9%, 0 %, and 0.1% for Subject 4; 93.5%, 3.5%, and 3.1% for Subject 5; 98.6%, 0.8%, and 0.6% for Subject 6; 99.3%, 0.7%, and 0.0% for Subject 7.

Acquisition

Imaging type(s)	functional MRI
Field strength	3T
Sequence & imaging parameters	fMRI data were collected using a 3.0-Tesla Siemens MAGNETOM Verio scanner. An interleaved T2*-weighted gradient-echo echo planar imaging (EPI) scan was performed to acquire functional images covering the entire brain (TR, 2000 ms; TE, 43 ms; flip angle, 80 deg; FOV, 192 × 192 mm; voxel size, 2 × 2 × 2 mm; slice gap, 0 mm; number of slices, 76; multiband factor, 4).
Area of acquisition	The imaging was done to acquire functional images covering the entire brain.

Diffusion MRI ☐ Used ☒ Not used

Preprocessing

Preprocessing software	We performed the MRI data preprocessing through the pipeline provided by FMRIPREP (version 1.2.1). For functional data of each run, first, a BOLD reference image was generated using a custom methodology of FMRIPREP. Using the generated BOLD reference, data were motion corrected using mcflirt from FSL (version 5.0.9) and then slice time corrected using 3dTshift from AFNI (version 16.2.07). This was followed by co-registration to the corresponding T1w image using boundary-based registration implemented by bbregister from FreeSurfer (version 6.0.1). The coregistered BOLD time-series were then resampled onto their original space (2 × 2 × 2 mm voxels) using antsApplyTrainsforms from ANTs (version 2.1.0) using Lanczos interpolation.
Normalization	The data were not normalized.
Normalization template	The data were not normalized.
Noise and artifact removal	A constant baseline, a linear trend, and six motion parameters were removed.
Volume censoring	No volume censoring was applied.

Statistical modeling & inference

Model type and settings	The data samples were temporally shifted by 4 s (2 volumes) to compensate for hemodynamic delays, were despiked to reduce extreme values (beyond ± 3 SD for each run), and were then averaged within each 8-s trial (training session, four volumes), the last 6-s period of each trial (test session, three volumes corresponding to second to fourth volumes in each trial).
Effect(s) tested	Multivoxel pattern regression models were constructed to predict (regress) feature values of a deep neural network model, from patterns of brain activity in tested brain areas.

Specify type of analysis: ☐ Whole brain ☒ ROI-based ☐ Both

Anatomical location(s)	We used brain areas functionally defined for individual subjects. V1, V2, V3, and V4 were delineated following the standard retinotopy experiment. The lateral occipital complex (LOC), fusiform face area (FFA), and parahippocampal place area (PPA) were identified using conventional functional localizers. A contiguous region covering the LOC, FFA, and PPA was manually delineated on the flattened cortical surfaces, and the region was defined as the higher visual cortex (HVC). Voxels overlapping with V1–V3 were excluded from the HVC. Voxels from V1–V4 and the HVC were combined to define the visual cortex (VC).

Statistic type for inference (See Eklund et al. 2016)	No voxel-wise or cluster-wise statistical modeling was performed.
Correction	No voxel-wise or cluster-wise statistical modeling was performed.

Models & analysis

n/a	Involved in the study
☒	☐ Functional and/or effective connectivity
☒	☐ Graph analysis
☐	☒ Multivariate modeling or predictive analysis

Multivariate modeling and predictive analysis	We used a set of linear regression models to construct multivoxel decoders to decode a deep neural network (DNN) feature pattern for a single presented image from a pattern of fMRI voxel values obtained in the training session (training dataset; samples from 6000 trials for each subject). The training dataset was used to train decoders to predict the values of individual units in feature patterns of all DNN layers (one decoder for one DNN unit). Decoders were trained using fMRI patterns in an entire visual cortex (VC) or individual visual subareas (V1–V4 and HVC), and voxels whose signal amplitudes showed the highest absolute correlation coefficients with feature values of a target DNN unit in the training data were provided to a decoder as inputs (with a maximum of 500 voxels).<br>The trained decoders were then applied to the fMRI data obtained in the test session (test dataset) to decode feature values of individual DNN units from fMRI samples constructed for each trial (samples from 160 single-image trials and 720 attention trials for each subject).<br>We further performed the image reconstruction analysis from the decoded feature values, in which pixel values of an input image were optimized based on a set of target (or decoded) DNN features such that the DNN features computed from the input image become closer to the target DNN features.
